# Effectiveness of an online education program for asthma patients in general practice: study protocol for a cluster randomized controlled trial

**DOI:** 10.1186/s12890-022-02217-2

**Published:** 2022-12-01

**Authors:** Stefanie Eck, Alexander Hapfelmeier, Klaus Linde, Konrad Schultz, Jochen Gensichen, Linda Sanftenberg, Thomas Kühlein, Stefanie Stark, Ildikó Gágyor, Christian Kretzschmann, Antonius Schneider, for the Bavarian Practice-Based Research Network (BayFoNet)

**Affiliations:** 1 Institute of General Practice and Health Services Research, TUM School of Medicine, Technical University of Munich (TUM), Orleansstraße 47, 81667 Munich, Germany; 2 Institute for AI and Informatics in Medicine, TUM School of Medicine, Technical University of Munich (TUM), Munich, Germany; 3 Clinic Bad Reichenhall, Center for Rehabilitation, Pneumology and Orthopedics, Bad Reichenhall, Germany; 4 Institute of General Practice and Family Medicine, Ludwig-Maximilians-Universität in Munich (LMU Munich), Munich, Germany; 5 Institute of General Practice, Friedrich-Alexander University Erlangen-Nürnberg (FAU), Erlangen, Germany; 6 Department of General Practice, Universitätsklinikum Würzburg (UKW), Würzburg, Germany

**Keywords:** Asthma, Patient education, Self-management, Asthma knowledge, Digital intervention, Cluster randomized controlled trial, Primary care

## Abstract

**Background:**

Asthma education programs (AEPs) have been shown to increase quality of life and reduce emergency treatments and hospital admissions. Despite the proven benefits, only a minority of asthma patients attend such programs. To increase the number of educated patients, an online education program (electronic AEP, eAEP) for asthma patients has been developed. The present study aims to investigate the effectiveness of the eAEP in terms of asthma knowledge, asthma control and emergency treatments in general practice settings.

**Methods:**

This is a cluster randomized controlled trial including 100 patients with bronchial asthma from 20 general practices in Bavaria, Germany. General practices will be randomly assigned to either the intervention or control group. Patients in the intervention group will receive access to the eAEP and instructions to complete this program within two weeks. Patients in the control group will receive usual care including a referral to face-to-face AEP (fAEP) by a certified primary care physician or a pulmonologist according to guideline recommendations. Furthermore, patients of both the intervention and control groups will be invited to a follow-up consultation in their general practice after completion of the eAEP and fAEP (three weeks and twelve weeks after study inclusion, respectively) to discuss any open issues. Outcomes for both groups will be assessed at baseline (t_0_), after two weeks (t_1_), three months (t_2_) and six months (t_3_). The primary outcome is the comparison of asthma knowledge gain between intervention and control groups after completion of the eAEP (two weeks after study inclusion) and fAEP (twelve weeks after study inclusion), respectively. Secondary outcomes include asthma control, frequency of emergency treatments, patient autonomy as well as attitudes towards asthma medication.

**Discussion:**

The results of the present trial will provide knowledge about the effectiveness of an online education program for asthma patients compared to usual care in primary care.

**Trial registration:**

German Clinical Trials Register (DRKS), DRKS00028805. Registered 22 April 2022.

**Supplementary Information:**

The online version contains supplementary material available at 10.1186/s12890-022-02217-2.

## Background

Bronchial asthma is an inflammatory respiratory disease affecting both children and adults. Symptoms are periodic and often vary over the course of day and night. Common asthma symptoms include coughing, wheezing, shortness of breath and chest tightness [1]. In most cases, asthma requires long-term management in order to achieve asthma control, reduce exacerbations as well as the risk of asthma-related death and persistent airflow limitation [1]. Essential components of therapy recommended by guidelines include appropriate assessment and monitoring of asthma, drug therapy, as well as patient education [1].

Patient education is a substantial part of non-drug therapy and provides knowledge and skills in order to improve patient self-management [1, 2]. Structured asthma education programs (AEPs) address essential topics such as correct inhaler use, self-monitoring of symptoms, peak flow assessment, a written action plan in case of asthma worsening and the importance of regular review by health care providers. Evidence has shown that AEPs advance health-related outcomes encompassing an improvement in quality of life, and reductions in hospital admissions and emergency treatments [3]. In Germany, AEPs need to be accredited in order to receive reimbursement for patient education in practices. In primary care, AEPs are predominantly provided in presence by pulmonologists or general practitioners (GPs) certified for conducting AEPs.

However, despite the nationwide existence of several structured patient education programs, only few patients with asthma participate in such programs [3, 4]. AEPs often entail barriers for patients as patients must be willing to invest time, practices offering AEPs involve long journeys or include limited capacity in scheduling. Additionally, COVID-19 has generated a considerable reduction in face-to-face appointments offered [5].

Due to improved accessibility, online formats might lower the threshold for participation in education programs and thereby increase participation rates. As a consequence, an online education program (electronic AEP, eAEP) for asthma patients was developed based on the face-to-face AEP (fAEP) of the Bad Reichenhall clinic in Germany, a center for inpatient pulmonary rehabilitation. The online format allows flexible use via PC, notebook or smartphone and enables patients to acquire knowledge on asthma basics, drug therapy as well as self-management.

The eAEP has already been evaluated in two proof-of-concept studies [6, 7]. It was first evaluated in 2018 in a randomized controlled pilot study conducted in the aforementioned pulmonary rehabilitation center in Bad Reichenhall, Germany. This study showed that a combination of eAEP and conventional AEP resulted in a significantly higher asthma knowledge compared to patients only attending the fAEP [6]. However, as GPs are of substantial importance in the management of patients with asthma, the eAEP could prove particularly helpful in primary care. Since the significance of the results of a rehabilitation clinic is limited when transferring to primary care, a single-arm pilot study was conducted in 12 general practices in Upper Bavaria, Germany, in 2019 [7]. After completion of the eAEP, patients received a questionnaire sent by mail consisting of an evaluation of the online program and an asthma knowledge test (AKT) [8]. The eAEP was well accepted and evaluated positively by patients of primary care. After completion of the eAEP a doubling of the asthma knowledge score in the AKT was observed (corresponding scale: 0 to 54 points) [7]. Consequently, the eAEP also proved to be feasible in primary care.

Nonetheless, the effectiveness of the eAEP at the level of general practice on asthma knowledge as well as its effect on additional clinical outcomes remain uncertain.

Moreover, asthma as a chronic disease commonly requires long-term management as well as self-management skills. As patients’ willingness and ability to engage in self-management may vary depending on multiple factors such as literacy, attitudes towards asthma and medications and desire for autonomy, patients’ individual expectations and beliefs should be highlighted [1]. We assume that patient education leads to higher preference for participation in shared decision making and increased medication adherence.

### Aims and Outcomes

This cluster randomized controlled trial (cRCT) aims to investigate the effectiveness of the eAEP compared to usual care in terms of 1) asthma knowledge, 2) asthma control, and 3) emergency treatments including the following endpoints:

1. Baseline-adjusted difference in the number of correctly answered questions in the AKT [8] between intervention and control groups
  - primary endpoint: after the eAEP (two weeks after study inclusion) and fAEP (twelve weeks after study inclusion), respectively.
  - secondary endpoint: three months after the eAEP (three months after study inclusion) and fAEP (six months after study inclusion), respectively.
  - secondary endpoint: at the end of the study (six-month follow-up).

Further secondary endpoints include:

2) Baseline-adjusted difference in the total sum score obtained in the asthma control test (ACT) [9] between intervention and control groups
  - three months after the eAEP (three months after study inclusion) and fAEP (six months after study inclusion), respectively.
  - at the end of the study (six-month follow-up).
3) Difference in frequency of emergency treatments (inpatient and outpatient treatments of asthma) between intervention and control groups at six months.

This trial further examines the relation between asthma knowledge and 4) patient autonomy as well as 5) attitudes towards asthma medication including the following endpoints:

4) Correlation between the total sum score achieved in the autonomy preference index (API) [10] at the time point baseline assessment and the change in correctly answered questions in the AKT after the eAEP and fAEP, respectively.
5) Correlation between the total sum score obtained in the questionnaire on attitudes towards asthma medication at the time point baseline assessment and the change in correctly answered questions in the AKT after the eAEP and fAEP, respectively.

## Methods/Design

### Study design

This study will be conducted as multicentre cRCT including 100 patients with bronchial asthma from 20 general practices in Bavaria, Germany. General practices will be randomly assigned to either the intervention or control group. Therefore, patients in one practice will receive the same treatment. Cluster randomization is a common procedure in health services research in primary care in order to prevent contamination of care [11–13].

**Fig. 1:**
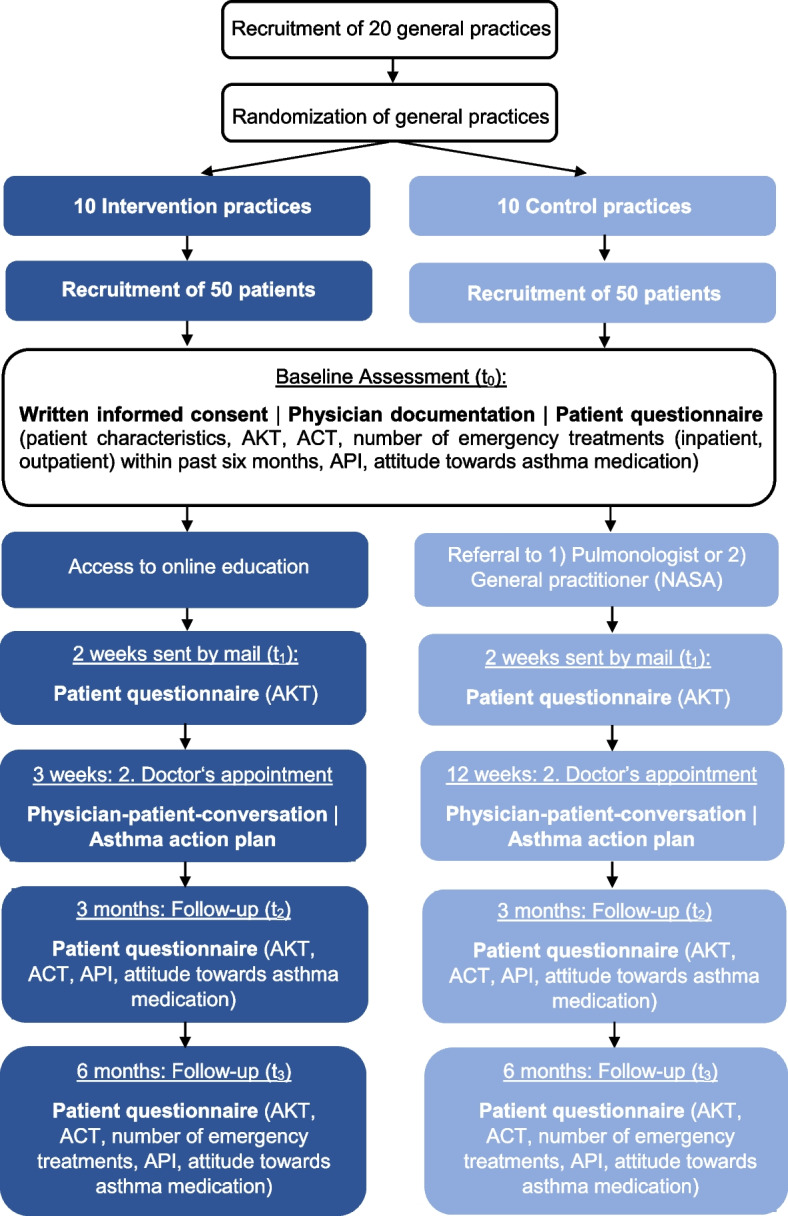
Overview of the course of the study. AKT implies asthma knowledge test; ACT, asthma control test; API, autonomy preference index; NASA, national asthma education program for adult asthmatics; t_0_, time point 0 (baseline assessment); t_1_, time point 1 (two weeks after study inclusion); t_2_, time point 2 (three-month follow-up); t_3_, time point 3 (six-month follow-up)

All patients with asthma will be informed about the study and informed consent will be obtained. Patients from general practices in the intervention group will receive free access to the eAEP and will be asked to complete the eAEP within the following two weeks. Patients from general practices in the control group will receive usual care including a referral to a practice of pulmonology or a certified general practice for structured fAEP. Patients will be required to attend the fAEP within twelve weeks. As part of the baseline assessment, patients will complete a questionnaire including questions on their asthma characteristics, current medication and emergency treatments within the past six months. In addition, a structured questionnaire including the AKT, ACT and API as well as questions on patients’ attitude towards asthma medication will be handed out (further details on the questionnaires are depicted in the section ‘Measurements’). Furthermore, current lung function parameters will be extracted from patient chart or spirometry will be performed to assess lung function in terms of FEV_1_ and FEV_1_/FVC. Two weeks after study enrolment, all patients will receive the AKT by mail to complete the questionnaire again. After completion of the eAEP (three weeks after inclusion) or fAEP (twelve weeks after inclusion) patients will attend a follow-up consultation in the general practice in order to review the asthma knowledge gain as well as to discuss any open issues. In order to ensure adherence to the second appointment, all patients will be contacted via phone by an employee of the Institute of General Practice and Health Services Research at the Technical University of Munich. As part of a follow-up, patients in both groups will be contacted again by mail after three as well as six months and asked to complete a questionnaire comprising the AKT, ACT, API as well as questions on the attitude towards asthma medication. In addition, at the six-month follow-up, participants will be inquired to answer questions about emergency treatments. A detailed overview of the course of the present trial can be seen in Fig. 1.

### Recruitment and Randomization

In this study, 20 general practices in Bavaria, Germany, will be included. Practices will be recruited by contacting GP’s of the institute’s teaching practices network and of the bavarian practice-based research network (BayFoNet). The Institute of General Practice and Family Medicine, Ludwig-Maximilians-University in Munich (LMU Munich) and the Institute of General Practice, Friedrich-Alexander University Erlangen-Nürnberg (FAU), which are also part of BayFoNet, will support recruitment of general practices. The allocation of the general practices to the intervention or control group will be conducted by a statistician (AH) of the study team, whom is not involved in the study conduct. For this purpose, a randomization sequence will be generated using the program R (The R Foundation for Statistical Computing, Vienna, Austria). The allocation ratio of intervention to control group at 1:1, no stratification, and randomly varying block sizes will be documented in advance. The randomization list will be prepared and retained by the statistician. After inclusion of general practices in the study, practices will be consecutively added to the randomization list by the statistician and assigned an appropriate sequential number. The statistician will report the corresponding classification of the practice into the study arm to the study coordinator.

After allocation of the general practice to the intervention or control group, patients will be consecutively recruited by the participating general practice. Patients will not be blinded with regard to the intervention. The practice team will check the inclusion and exclusion criteria. Furthermore, the attending physician will inform patients about the study. If patients may be eligible for the study but are unwilling to participate, age, sex, and the reason for non-participation will be documented anonymously using a non-responder list. Thus, it is possible to conduct a non-responder analysis to assess acceptance of the eAEP offer as well as fAEP.

### Characteristics of participants

#### Inclusion criteria

Adult patients with confirmed diagnosis of bronchial asthma visiting one of the 20 participating practices will be included consecutively. All patients had been diagnosed with asthma by a pulmonologist according to defined criteria. Patients are eligible for inclusion if they have not yet attended an asthma education program or have not attended such program within the past ten years. Patients must also declare their written informed consent to participate in the study.

#### Exclusion criteria

Patients with the following criteria will be excluded:

- Patients not agreeing to the study
- Patients under the age of 18 years due to legal grounds
- Patients not having sufficient knowledge of German as the online education program is currently only available in German
- Patients whose asthma education dates back ≤ ten years

### Interventions

**Fig. 2:**
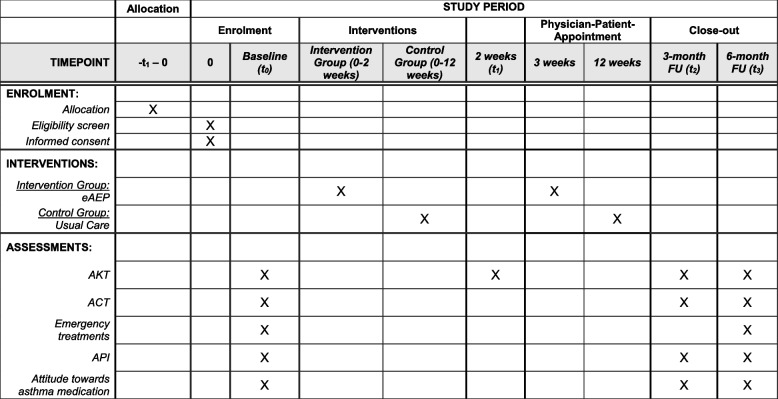
Standard Protocol Items: Recommendations for interventional trials (SPIRIT) schedule. eAEP implies electronic asthma education program; AKT, asthma knowledge test; ACT, asthma control test; API, autonomy preference index; FU, follow-up; t_0_, time point 0 (baseline assessment); t_1_, time point 1 (two weeks after study inclusion); t_2_, time point 2 (three-month follow-up); t_3_, time point 3 (six-month follow-up)

Patients’ schedule of enrolment, interventions and assessments is summarized in Figure 2.

#### Intervention Group

Patients from general practices in the intervention group will receive access to the eAEP. Patients will also obtain a peak flow meter and an asthma diary from their GP in advance. The use and application of these tools is addressed in the eAEP. The eAEP consists of a webpage and is available at www.asthmaselbstmanagement.de. It is intended that patients work through the eAEP independently at home using a PC or notebook. The eAEP encompasses an introductory page, four chapters including a short quiz at the end of each chapter, a final quiz after completing the courses as well as a glossary with frequently asked questions. Chapter one conveys basic knowledge on bronchial asthma as a chronic disease. Furthermore, it provides information on the structure and function of the respiratory tract. In chapter two, patients will be taught about asthma medication and its application. Chapter three is about self-management and focuses on the application of an asthma diary, an asthma action plan, as well as the peak flow measurement. The last chapter provides information on how to behave during an asthma emergency. After completing the four chapters, patients’ asthma knowledge will be tested in the final quiz and feedback on their performance for sufficient learning will be provided. If asthma patients answered fewer than 80 percent of the questions correctly, they will be advised to work through the courses again.

Patients do not enter any personal data when performing the eAEP. Furthermore, there is no documentation on how often and for how long the website was accessed. In total, two to three hours are estimated for the complete and conscientious use of the program.

The eAEP is based on the structured and evaluated fAEP of the Bad Reichenhall clinic in Germany. The eAEP was developed by a pneumologist with long-time experience in patient education (KS) and a general practitioner (AS). Both developers are members of the national asthma guideline board [2].

Three weeks after study enrolment, patients in the intervention group will attend the follow-up appointment in the general practice to ensure that the content of the eAEP had been understood correctly as well as to ensure patient safety. By using the “physician-patient-appointment” questionnaire, GPs will test patients’ asthma knowledge (see section ‘Measurements’).

#### Control Group (usual care)

Patients from general practices in the control group will receive care as usual according to guideline recommendations. This encompasses either a referral to a pulmonary practice for structured fAEP or a structured fAEP in a certified general practice. Patients will be asked to attend the fAEP within twelve weeks. Furthermore, patients in the control group will also receive a peak flow meter and an asthma diary in advance.

Twelve weeks after study enrolment, patients in the control group will visit the general practice again to discuss remaining questions as well as to test patients’ asthma knowledge on the basis of the “physician-patient-appointment” questionnaire. At the end of the study, patients of the control group will also receive access to the eAEP.

### Measurements

#### Physician documentation at baseline assessment (time point 0 (t_*0*_))

After obtaining written informed consent, the respective physician will provide information on current asthma medication of the patient as well as asthma symptoms over the past four weeks. In addition, lung function in terms of FEV_1_ and FEV_1_/FVC will be assessed by performing a spirometry or respective parameters will be extracted from the patient chart (see Additional file 1 for further details).

#### Patient questionnaire at baseline assessment (t_*0*_)

Immediately after inclusion in the study, patients will be asked to complete a questionnaire encompassing questions on their asthma characteristics, the AKT, ACT, API as well as questions on patients’ attitude towards asthma medication and emergency treatments.

#### Asthma Knowledge Test (AKT)

The AKT is a questionnaire assessing asthma knowledge and self-management skills regardless of disease severity [8]. This questionnaire is particularly suitable for quality assurance and evaluation of asthma education programs. It encompasses four subdomains including knowledge regarding pathology (7 items), drug treatment (15 items), self-management (25 items) as well as non-drug interventions (7 items). The maximum score is 54. Since the AKT is not a fixed instrument, it must be adapted to current guidelines. Consequently, two of 56 items were removed and thus the version applied in the current study consists of 54 questions. Furthermore, the original version includes the answer options “correct” and “incorrect” [8]. In order to reduce the proportion of unanswered questions, the additional answer option "don't know" was included after consultation with the authors.

#### Asthma Control Test (ACT)

The ACT is a validated, frequently used questionnaire to evaluate symptom control of patients with bronchial asthma [9]. Patients will answer five questions regarding symptoms during day and night, the use of emergency medication, as well as self-assessment of asthma control over the past four weeks. The maximum score is 25. Depending on the achieved sum score, patients’ asthma will be classified into well controlled, poorly controlled and very poorly controlled.

#### Autonomy Preference Index (API)

The API assesses patients’ preference regarding information (8 items) as well as involvement in medical decisions (6 items) [10]. Each item uses a five-point scale with the lowest preference scored 1 and the highest preference scored 5. Sum scores will range from 0 to 100, with 100 corresponding to the strongest possible desire for information as well as involvement in medical decisions. The API was translated into German and was validated [14].

#### Attitude towards asthma medication

In order to assess the attitude towards medication for bronchial asthma, eight statements on drug therapy will be evaluated by the patient, given in Table 1. The following options are available for assessing the statements: (1) "Strongly disagree", (2) "Some of the time", (3) "Most of the time", (4) "Strongly agree". If one of the drugs mentioned in the statements was not prescribed, the statement is marked by the patient with (0) "Drug was not prescribed" (see Additional file 2). The items are summed up accordingly.

**Table 1:** Statements of the questionnaire regarding attitude towards asthma medication

Statements	
1	I use the reliever spray every day.
2	I use the reliever spray no more than twice per week.
3	I take inhaled corticosteroids every day.
4	I take inhaled corticosteroids when needed.
5	I use a combination inhaler every day.
6	I use a combination inhaler when needed.
7	I take as little asthma medication as possible, even if I have asthma symptoms.
8	I manage my asthma symptoms well in everyday life.

#### Emergency treatments

In order to record emergency treatments due to asthma attacks, patients will answer two questions. These include the number of inpatient as well as outpatient emergency treatments over the past six months.

#### Patient questionnaire two weeks after study inclusion (t_*1*_)

All patients will receive a questionnaire by mail two weeks after study inclusion. Patients in the intervention group will be asked to complete the AKT again after they have completed the eAEP. Patients in the control group will also receive the AKT after two weeks, even if no fAEP has been attended by this time. Thus, it will be assessed whether they have already acquired knowledge about the disease independently of a fAEP using other sources such as the internet. In addition, patients from general practice in the intervention group will be asked about their internet use in general and whether they had participated in a fAEP in addition to completing the eAEP.

#### Physician-patient-appointment after completion of the eAEP and fAEP (three and twelve weeks after study inclusion, respectively)

After completion of the eAEP as well as fAEP, essential contents of the education programs will be reviewed together with the GP using a questionnaire based on the national asthma care guideline (see Additional file 3 for further details). This final appointment is of high importance in order to verify the acquired knowledge of the patients. Therefore, an employee of the Institute of General Practice and Health Services Research at the Technical University of Munich will contact the patient by phone to ensure that the GP appointment is attended.

#### Follow-up at three (t_*2*_) and six months (t_*3*_)

After three and six months, the sustainability of the acquired asthma knowledge will be tested. For this purpose, patients receive a questionnaire by mail including the AKT, ACT and API as well as questions on the attitude towards asthma medication. Moreover, patients from general practices of the intervention group will be asked whether they have attended a fAEP in addition to the eAEP. Additionally, patients will answer questions regarding emergency treatments over the past six months at the six-month follow-up.

### Data Management and Monitoring

After informed consent is obtained and the data protection declaration is signed, the patient will be assigned a pseudonymized study ID. Further data and examination results are documented and stored under this ID. For instance, neither the name, nor the initials, nor the exact date of birth will appear in the identification number. The patient identification list remains at the Institute of General Practice and Health Services Research and will only be accessible to authorized study personnel. The data collection process as well as the study procedures will be supervised by a research associate.

### Risks

There are no immediate risks. However, it cannot be excluded that the content of the eAEP may be misunderstood in individual cases. Therefore, the follow-up review at the general practices will take place. As described in section ‘Measurements’, this second doctor’s appointment is of high importance in order to review the knowledge acquired by the patient. A reminder via phone will ensure that the patient attends this appointment.

### Statistics

#### Sample size estimation

The power calculation is based on the results of the pilot study conducted in 2019 [7] using a two-sample t-test: An average difference of 22 points in the AKT (corresponding scale ranges from 0 to 54 points) was observed between baseline measurement and after completion of the eAEP (two weeks after study inclusion). A more conservative approach of an average difference of 20 points is applied for planning the current study. As the pilot study did not include a control group, an average asthma knowledge gain of 5 points was estimated for the control group of the present study. This estimation is based on the assumption, that about 25% of the patients in the control group will attend a fAEP and therefore achieve an average knowledge gain of maximum 20 points [3, 4]. The remaining 75% of the patients are expected to reach an average knowledge gain of 0 points. This results in 0.25 x 20 points + 0.75 x 0 points = 5 points. In order to include all available data in statistical analyses as well as to prevent an attrition bias, the intention-to-treat principle is applied in this study with an expected drop-out rate of 20%. Performing a worst-case-analysis [15], it is assumed that drop-out patients in the intervention group achieve an average knowledge gain of 0 points. Accordingly, an average asthma knowledge gain of 16 points is expected in the intervention group (0.80 x 20 points + 0.20 x 0 points). Considering a worst-case-analysis, the average asthma knowledge gain is assumed to double for drop-out patients in the control group resulting in an average knowledge gain of 6 points (0.80 x 5 points + 0.20 x 10 points). The sample size estimation is based on a standard deviation of 15. Using a two-sample t-test and a two-sided 5 % significance level, the number of patients per group is 36 to achieve a power of 80 %. Due to cluster randomization, the number of patients has to be increased by the design effect (DE). The sample size estimation of the current study is based on an intracluster correlation coefficient (ICC) of 0.10 [16], a mean cluster size of 5 and a resultant DE of 1.4 (calculation based on [13]). Regarding the primary outcome asthma knowledge gain, the number of patients required is therefore 100 (2 x 36 x 1.4 ≈ 100).

#### Statistical analysis

Descriptive statistics are used to describe the data included for the statistical analysis and are reported as the mean, standard deviation, median, quartile and ranges as well as absolute and relative frequencies. The primary endpoint is the asthma knowledge gain after the eAEP (two weeks after study inclusion) and fAEP (twelve weeks after study inclusion), respectively. A confirmatory hypothesis test for difference between intervention and control group is performed by a linear mixed-effects model at a two-sided 5% significance level. Based on the intention-to-treat principle, all patients will be included in analysis. The baseline value (number of correctly answered questions in the AKT at baseline), time since eAEP/fAEP, and group allocation (intervention or control group) will be used as fixed effects. Cluster effects of the general practices will be included in analyses as random intercepts. As previously mentioned, missing data for this variable will be imputed by performing a worst-case-analysis. Thereby, the average knowledge gain for drop-out patients is expected to be 0 points in the intervention group and 10 points in the control group. A comparison of the asthma knowledge gain between intervention and control group will also be made three months after completion of the eAEP (three months after study inclusion)/fAEP (six months after study inclusion) and at the end of the study (six-month follow-up). Similar calculations will be applied for the secondary outcome asthma control (difference in the achieved sum score of the ACT three months after completion of the eAEP/fAEP as well as at the six-month follow-up) (see Table 2). Emergency treatments as a secondary outcome will be considered binary in the analyses (0: No; 1: Yes, at least one emergency treatment). Due to the consideration of measurement repetition as well as data grouped in clusters, generalized estimating equations (GEE) will be applied. Group allocation is used as a covariate. Spearman or Pearson correlation coefficients will be calculated for the relation between 1) patients’ autonomy preference and asthma knowledge as well as between 2) patients’ attitude towards asthma medication and asthma knowledge. All analyses will be conducted exploratively with and without imputation in terms of a worst-case-analysis.

**Table 2:** Overview of primary and secondary endpoints of the respective outcomes for linear mixed-effects models

			Time point (t)	
Outcomes			Intervention group	Control group
Asthma knowledge	Primary endpoint	AK gain **after** eAEP / fAEP	Two weeks after study inclusion	Twelve weeks after study inclusion
	Secondary endpoints	AK gain **three months** after eAEP / fAEP	Three months after study inclusion	Six months after study inclusion
		AK gain **at the end of the study**	Six months after study inclusion	Six months after study inclusion
Asthma control	Secondary endpoints	Total sum core in the ACT **three months after** eAEP / fAEP	Three months after study inclusion	Six months after study inclusion
		Total sum core in the ACT **at the end of the study**	Six months after study inclusion	Six months after study inclusion

*Annotation:* Planned model for confirmatory hypothesis test for difference between intervention and control group: Linear mixed-effects model (5% significance level). *AK* implies asthma knowledge, *eAEP* electronic asthma education program, *AEP* asthma education program, *ACT* asthma control test

## Discussion

The present cRCT aims to investigate the effectiveness of an online asthma education program compared to usual care in terms of asthma knowledge, asthma control and emergency treatments in primary care settings.

The online character of the education program presents a promising opportunity for asthma patients to increase their asthma knowledge by virtue of providing several benefits over conventional fAEPs. Particularly, improved accessibility to information is a key advantage of digital interventions as they may be extremely appealing for patients by lowering barriers to participate primarily in terms of cost and time effectiveness [17–19]. However, evidence supports the application of a multidisciplinary approach for effective asthma management. Besides education, this includes the provision of an asthma action plan and the support of regular reviews by a physician for improving self-management skills and therapy adherence [3]. This approach has already been included in the randomized trial by van der Meer and colleagues [20]. The results showed that an internet-based self-management intervention including asthma control monitoring, online and group education as well as medical review yielded improvements in asthma control and lung function. This highlights the importance for asthma patients of the present study to attend the second doctor’s appointment, discuss any open issue regarding the chronic disease and download essential self-management tools. In order to ensure attendance in the present study, patients will be reminded of their second appointment by means of a phone call.

Although only 60 percent of patients in the pilot trial conducted in 2019 attended this appointment, the study still showed that completion of the eAEP resulted in a great increase in asthma knowledge. Patients’ knowledge levels also remained stable after three and six months [7]. However, the significance of the results may be limited due to the pilot character of the study. Therefore, it is reasonable to conduct a confirmatory, cRCT that includes a control group performing usual care and an additional assessment of clinical outcomes such as asthma control and emergency treatments.

Nonetheless, a common and well-known disadvantage of cRCTs may be the differences in recruitment between intervention and control groups [21]. In most cases, the allocation to the intervention group might be more attractive leading to an increased recruitment of patients and thereby evoking a risk of selection bias. However, a major advantage of cRCTs is the avoidance of contamination of care. Therefore, it is most reasonable to select this study design as it allows to estimate the impact of the eAEP compared to usual care. Furthermore, it is likely that patients of younger age as well as higher education level are more prone to participate in this study as it involves engagement in an online program and thereby requiring skills and knowledge in internet applications. However, the pilot study in 2019 has shown that patients were comparatively older with a mean age of 48 years. Furthermore, it has been shown that patients had a rather standard education [7]. In the present study, the asthma knowledge of patients of both the intervention as well as control groups will be assessed two weeks after study enrolment. While it is assumed that patients of the intervention group have already completed the eAEP by this time, it is rather unlikely that patients of the control group will be able to attend a fAEP immediately after referral. Nevertheless, we aim to investigate the asthma knowledge gain within the context of clinical reality. Another challenge to face might be the repeated measurement of asthma knowledge using the same questionnaire. Applying the same knowledge test several times could result in an increase in correctly answered questions and thereby in a better performance. Nevertheless, as this questionnaire involves 54 items addressing a great variety of topics, this phenomenon seems rather unlikely to happen. In addition, the fact that the eAEP is only available in German so far, could present a further limitation. It prevents patients incapable of understanding and speaking German from participating in the study. Therefore, the study population may not be representative of the general population and thereby the generalizability of the results may be limited. However, it is assumed that most of the asthma patients visiting general practices are eligible to participate in the study as there are only few exclusion criteria included.

The results of the cRCT may promote the implementation of an online education program for asthma patients in combination with the application of an asthma action plan and regular reviews by a physician in primary care.

## Supplementary Information

**Additional file 1.** Physician documentation at baseline assessment.

**Additional file 2.** Questionnaire on attitudes towards asthma medication.

**Additional file 3.** Physician documentation at physician-patient-appointment after completion of the online asthma education program and face-to-face asthma education program.

## Data Availability

The created dataset will be available from the corresponding author on reasonable request.

## Abbreviations

AEP: Asthma education program
ACT: Asthma control test
AKT: Asthma knowledge test
API: Autonomy preference index
BayFoNet: Bavarian practice-based research network
cRCT: Cluster randomized controlled trial
DE: Design effect
eAEP: Electronic asthma education program
fAEP: Face-to-face asthma education program
FEV_1_: Forced expiratory volume in one second
FVC: Forced vital capacity
GEE: Generalized estimating equations
GP: General Practitioner
ICC: Intracluster correlation coefficient
t_0_: Time point 0
t_1_: Time point 1
t_2_: Time point 2
t_3_: Time point 3
