# Supplementary material for: Effectiveness of an online education program for asthma patients in general practice: study protocol for a cluster randomized controlled trial
Source: BMC Pulm Med. 2022 Dec 1;22:457. doi: 10.1186/s12890-022-02217-2 (PMC9713723; doi:10.1186/s12890-022-02217-2)
Supplement: Supplementary file 1 — Additional file 1. Physician documentation at baseline assessment. [file 12890_2022_2217_MOESM1_ESM.docx]

**Physician Documentation**

**Medication of bronchial asthma:**

None

Short-acting beta-2 agonists

Inhaled glucocorticosteroid

Inhaled long-acting beta-2-agonists, LABA

Systemic glucocorticosteroids

Other: _____________________________________

**Information on the patient’s bronchial asthma within the past four weeks:**

Symptoms more than twice a week during the day

Nocturnal awakening due to asthma

Use of reliever spray* for symptoms more than twice per week

Activity limitation due to asthma

*excluding reliever spray used prior to physical activity

**Lung function:** *Please fill in current values.*

**FEV_1_ (%): _______**

**FEV_1_/FVC (%): _______**

**_____________________ _______________________**

**Physician Date/Signature**

**General information**

Patient education is an essential part of asthma management. However, many patients with asthma have not participated in education programs. In order to provide access to education programs for as many patients as possible, an online education program for asthma patients has been developed. In this study, patients will be assigned to either a test group that receives online access or a control group that attends a convential face-to-face asthma education program.

*Please explain the purpose of the study to the patient and obtain written informed consent as well as the data protection declaration.* ***Please also perform a lung function test (spirometry)*** *and fill in the values on page 1.*
