# Supplementary material for: Effectiveness of an online education program for asthma patients in general practice: study protocol for a cluster randomized controlled trial
Source: BMC Pulm Med. 2022 Dec 1;22:457. doi: 10.1186/s12890-022-02217-2 (PMC9713723; doi:10.1186/s12890-022-02217-2)
Supplement: Supplementary file 2 — Additional file 2. Questionnaire on attitudes towards asthma medication. [file 12890_2022_2217_MOESM2_ESM.docx]

**Attitudes towards asthma medication**

Treating bronchial asthma with medication is a challenge for many patients. Some sprays have to be taken daily. Others only when one’s condition is acute. As a rule, these include:

- **inhaled corticosteroids** (often in a red, orange or brown inhaler),
- **reliever spray** (= bronchodilator medication that acts quickly, often in a blue inhaler),
- or a **combination inhaler** (combines cortisone and a reliever spray)

Using the following questions, we would like to discern your ability to cope with your asthma sprays. Please place only one cross in each line. If you were not prescribed any of the following drugs, please mark this with “Drug was not prescribed”.

|  | Drug was not prescribed | Strongly agree | Most of the time | Some of the time | Strongly disagree |
| --- | --- | --- | --- | --- | --- |
| I use the reliever spray every day. |  |  |  |  |  |
| I use the reliever spray no more than twice per week. |  |  |  |  |  |
| I take inhaled corticosteroids every day. |  |  |  |  |  |
| I take inhaled corticosteroids when needed. |  |  |  |  |  |
| I use a combination inhaler every day. |  |  |  |  |  |
| I use a combination inhaler when needed. |  |  |  |  |  |
| I take as little asthma medication as possible, even if I have asthma symptoms. |  |  |  |  |  |
| I manage my asthma symptoms well in everyday life. |  |  |  |  |  |
