# Supplementary material for: Effectiveness of an online education program for asthma patients in general practice: study protocol for a cluster randomized controlled trial
Source: BMC Pulm Med. 2022 Dec 1;22:457. doi: 10.1186/s12890-022-02217-2 (PMC9713723; doi:10.1186/s12890-022-02217-2)
Supplement: Supplementary file 3 — Additional file 3. Physician documentation at physician-patient-appointment after completion of the online asthma education program and face-to-face asthma education program. [file 12890_2022_2217_MOESM3_ESM.docx]

**Dear colleague,**

Following the online training (for control group: conventional asthma education program) conducted by the patient, we would like to ask you to use this checklist to test your patient’s knowledge about asthma. We want to thereby ensure that the patient hast correctly understood the most important contents of the training program.

**Thank you for your participation!**

*During the patient interview, please guide yourself with the following questions and indicate whether the expectation was met.*

1. **What do you consider to be the most important medication for asthma therapy?**

*Expectation: Inhaled corticosteroids (ICS), salbutamol if needed*

**yes no**

|  |  |  |
| --- | --- | --- |

- ICS mentioned as controller?

|  |  |  |
| --- | --- | --- |

- Salbutamol/Bronchodilators mentioned as emergency spray/reliever?

1. **How do these drugs act?**

*Expectation: Anti-inflammation by ICS, bronchodilation by bronchodilators*

**yes no**

|  |  |  |
| --- | --- | --- |

- Could explain mechanism of action for ICS?

|  |  |  |
| --- | --- | --- |

- Could explain mechanism of action for bronchodilators?

1. **How can you identify a deterioration?**

*Expectation: Shortness of breath, deterioration of PEFs*

**yes no**

|  |  |  |
| --- | --- | --- |

- Shortness of breath mentioned?

|  |  |  |
| --- | --- | --- |

- PEF mentioned?

1. **What do you do when you feel worse?**

*Expectation: Pursed lip breathing, 2-4 strokes of short-acting spray, cortisone tablets if needed* **yes no**

|  |  |  |
| --- | --- | --- |

- Pursed lip breathing mentioned?

|  |  |  |
| --- | --- | --- |

- Short-acting spray mentioned?

|  |  |  |
| --- | --- | --- |

- Cortisone tablets mentioned?

1. **Demonstration of inhalation technique**

yes no

|  |  |  |
| --- | --- | --- |

- Inhalation technique demonstrated correctly?

|  |  |  |
| --- | --- | --- |

1. **PEF measurement discussed with patient? yes no**
2. **Personal asthma action plan completed with the patient? yes no**

|  |  |  |
| --- | --- | --- |

________________________ _________________________

**Physician Date/Signature**
